# Antimicrobial resistance profiles and associated factors of *Acinetobacter* and *Pseudomonas aeruginosa* nosocomial infection among patients admitted at Dessie comprehensive specialized Hospital, North-East Ethiopia. A cross-sectional study

**DOI:** 10.1371/journal.pone.0257272

**Published:** 2021-11-15

**Authors:** Habtamu Mekonnen, Abdurahaman Seid, Genet Molla Fenta, Teklay Gebrecherkos

**Affiliations:** 1 Department of Medical microbiology, Amhara Public Health Institute, Dessie branch, Dessie, Ethiopia; 2 Department of Medical Microbiology, College of Medicine and Health Science, Wollo University, Dessie, Ethiopia; 3 Department of Medical Microbiology, College of Medicine and Health Sciences, University of Gondar, Gondar, Ethiopia; Indian Institute of Technology Delhi, INDIA

## Abstract

**Introduction:**

Hospital admitted patients are at increased risk of nosocomial infections (NIs) with multi-drug resistant (MDR) pathogens which are prevalent in the hospital environment. *Pseudomonas aeruginosa* (*P*. *aeruginosa*) and *Acinetobacter baumannii* (*A*. *baumannii*) are common causes of NIs worldwide. The objective of this study is to determine antimicrobial resistance profiles and associated factors of *Acinetobacter* spp and *P*. *aeruginosa* NIs among hospitalized patients.

**Methods:**

A cross-sectional study was conducted at Dessie comprehensive specialized hospital, North-East Ethiopia, from February 1 to April 30, 2020. A total of 254 patients who were suspected of the bloodstream, urinary tract, or surgical site nosocomial infections were enrolled consecutively. Socio-demographic and other variables of interest were collected using a structured questionnaire. Specimens were collected and processed following standard microbiological procedures. Antimicrobial susceptibility was determined using the Kirby-Bauer disk diffusion method following Clinical and Laboratory Standards Institute guidelines. Data were analyzed with SPSS version 23 and p-value < 0.05 was considered statistically significant.

**Results:**

Overall, 13% of patients had nosocomial *Acinetobacte*r spp and/or *P*. aeruginosa infections. The culture positivity rate was 16(6.3%) for *Acinetobacte*r spp and 18(7.1%) for *P*. *aeruginosa*. Patients admitted in the surgical ward (Adjusted odds ratio (AOR):10.66;95% confidence interval (CI):1.22–93.23), pediatric ward (AOR:14.37;95%CI:1.4–148.5), intensive care unit (AOR:41.93;95%CI:4.7–374.7) and orthopedics (AOR:52.21;95%CI:7.5–365) were significantly at risk to develop NIs compared to patients admitted in the medical ward. Patients who took more than two antimicrobial types at admission were 94% (AOR:0.06; 95% CI:0.004–0.84) times more protected from NIs compared to those who did not take any antimicrobial. About 81% of *Acinetobacter* spp and 83% of *P*. *aeruginosa* isolates were MDR. Amikacin and meropenem showed promising activity against *Acinetobacter spp* and *P*. *aeruginosa* isolates.

**Conclusion:**

The high prevalence of MDR Acinetobacter *spp* and *P*. *aeruginosa* nosocomial isolates enforce treating of patients with NIs based on antimicrobial susceptibility testing results.

## Introduction

Nosocomial infection (NI) is defined as healthcare-associated infection (HCAI) developing at least 48–72 hr after admission or up to 3 days after discharge and was not incubating on admission [1]. According to the world health organization (WHO) fact sheet report, NI is a major global health problem affecting a hundred million patients annually [2] and rapidly increasing especially in developing countries [3]. The point prevalence of IN ranged from 5.7% to 19.1% and 3.5% to 12% in Low-and Middle-Income Countries (LMICs) and developed nations, respectively [2]. In Ethiopia, according to a recent meta-analysis, HCAI affected 16.96% of patients. Surgical site infection, urinary tract infection, bloodstream infection, and respiratory tract infection were the commonest types of HCAI in the country [4]. Besides associated mortality and morbidity, NI increases the occurrence of antimicrobial resistance [5], hospitalization times, and healthcare cost for the patients as well as the health care systems [6].

Bacterial pathogens could be acquired either exogenously or endogenously and are transmitted through direct or indirect contact between patients, healthcare workers, visitors, and contaminated objects or different hospital environmental sources. Risk factors of NIs are so diverse and depend on infection sites. The use of invasive devices [7, 8] and prior use of broad-spectrum antimicrobials increase the risk of infection [7]. Longer hospitalization [9], intravascular catheterization [10], age of the patient [7, 11], gender, surgery after admission, urinary catheterization [8], and type of hospital settings [11] were also identified as significant risk factors for nosocomial infections.

Epidemiological studies revealed that different species of both gram-positive and gram-negative bacteria are associated with NIs [12–15]. Among gram-negative pathogens, *A*. *baumannii* and *P*. *aeruginosa* have got significant importance associated with NI [12]. Recently in Nepal, *A*. *baumannii* (44.0%), and *P*. *aeruginosa* (40.1%) were the most common gram-negative bacteria isolated among hospitalized patients [2]. According to a recent meta-analysis report, the pooled proportion of *A*. *baumannii* associated NIs was 15.3% with the highest burden (20.9%) in the intensive care unit (ICU). Authors conclude that *A*. *baumannii* infection accounted for a significant risk to the health of hospitalized patients in Europe, Eastern Mediterranean regions, and Africa [16].

Both *P*. *aeruginosa* and *Acinetobacter* spp. are ubiquitous in hospital environments and frequently associated with NIs that are difficult to treat. This is because these organisms possess inherent drug resistance mechanisms (e.g. constitutive expression of AmpC beta-lactamases and efflux pumps, low permeability of the outer membrane, etc.). They are also gifted to acquire additional resistance mechanisms to multiple classes of antimicrobials (e.g. beta-lactams, aminoglycosides, and fluoroquinolones) through horizontal gene transfer [2, 17, 18]. Such behavior is responsible to resist multiple antimicrobials at the same time and limits the choice of antibiotics for the treatment of infections due to these microorganisms.

Infection due to multi-drug resistant (MDR) bacteria is challenging for treatment and resulted in poor clinical outcomes, prolonged hospitalization, and elevated health care costs [19, 20]. MDR *Acinetobacter* species and *P*. *aeruginosa* are recognized in the WHO lists as dangerous pathogens. The WHO has also ranked carbapenem-resistant *A*. *baumannii* as a critical priority pathogen that needs drug research and development [21]. Infections due to these microorganisms are a global health threat and are considered as the number one critical priority pathogen for which new therapeutics are urgently required [22]. In Ethiopia, scholars reported that more than half of isolated gram-negative rods were extended-spectrum beta-lactamase (ESBL) producers and 25% of isolates were resistant to carbapenem [13]. Motbainor et al [23] and Alemayehu et al [14] recently identified that all nosocomial isolates of *A*. *baumannii* and *P*. *aeruginosa* were MDR [23], and infection due to these bacteria is alarming in the country since it is difficult and impossible to manage with currently available antibiotics.

Despite the global increasing incidences of NIs associated with *Acinetobacter* spp. and *P*. *aeruginosa*, and the increasing frequency of antibiotic-resistant strains, there is limited data about the nosocomial importance of these bacterial species in Ethiopia [23]. In most clinical settings of the country, clinicians lack real and up-to-date information about the nosocomial importance of these organisms. This is mainly due to poor laboratory facilities and infrastructure to isolate these species and screening of drug-resistant strains. And also, factors associated with NIs due to *Acinetobacter* spp. and *P*. *aeruginosa* are not well identified in Ethiopia.

Moreover, the type of bacterial species colonizing the hospital environment changes over time and varies among hospitals within the country and different locations in the same hospital. It is also known that bacterial antibiotic resistance is a dynamic process so that resistance patterns observed in the past might not reflect the current situation. As a result, investigation of the causative agents and their current antimicrobial susceptibility profile is essential to optimize the management and reduction of the rate of NIs. The aim of this study is, therefore, to determine the prevalence and antimicrobial resistance pattern, and to identify factors associated with *Acinetobacter* spp. *and P*. *aeruginosa* infection among admitted patients suspected of nosocomial infection at Dessie comprehensive specialized hospital, Ethiopia.

## Materials and methods

### Study design, period, and setting

A hospital-based cross-sectional study was conducted from February 1 to April 30, 2020, at Dessie Comprehensive Specialized Hospital (DCSH), south wollo zone of Amhara Regional State, North-East Ethiopia. DCSH is found in Dessie town. The town has a total area of 15.08km^2^ and is located at 401 km far from the capital city of the county, Addis Ababa, and 471 km far from Bahir Dar, the capital city of Amhara regional state. The hospital provides specialized and referral services for more than 7 million people living in the catchment area. The hospital has 600 beds with eight major wards including Medical, Surgical, Obstetrics, Gynecology, Malnutrition, ICUs, and Pediatric wards for inpatient services. The hospital has an average daily patient flow of 600 visiting different outpatient departments (OPD) including adult OPD, Paediatric OPD, Emergency OPD, tuberculosis & HIV OPD.

### Patient selection

The source population was all hospital admitted patients during the study period. Patients who were admitted for more than 48 hrs and developed clinical evidence of nosocomial wound (surgical site) infections, bloodstream infection, and urinary tract infection were included in the study. The criteria of the European Centre for Disease Prevention and Control were used to define NIs and to select eligible study participants [24]. Patients who had either purulent drainage, pain, localized swelling, redness, or heat in the skin, subcutaneous tissue, deep soft tissue, organ or spaces, and one positive culture for Acinetobacter spp and *P*. *aeruginosa* after 48 hr of operation were considered as nosocomial surgical site infection. Patients who had either fever (> 38°C), urgency, frequency, dysuria, or suprapubic tenderness with no other recognized cause but has ≥10^2^ CFU/milliliters (ml) and ≥ 10^5^ CFU/ml of urine culture for catheterized and non- catheterized patients, respectively after 48 hr of admission were considered as nosocomial urinary tract infection. On the other hand, patients who had either fever (> 38°C), chills, or hypotension and one positive blood culture for *A*. *baumannii* and *P*. *aeruginosa* after 48 hr of admission were considered as nosocomial bloodstream infection and included as a study participants. Moreover, attending internists and surgeons were consulted for their decision if we were not able to select based on the above criteria. However, those patients who were unable to give clinical samples due to different conditions were excluded from the study.

### Sample size and sampling procedures

The minimum sample size was determined using the single population proportion formula: N = z^2^ p (1- p)/ d^2^, where N = minimum sample size; Z = standard normal distribution value at 95% CI which is 1.96; P = 8.4% prevalence of *A*. *baumannii* and *P*. *aeruginosa* infection taken from the previous study [23]; d = the margin of error taken as 4%. Accordingly, the estimated sample size was 185. However, 254 admitted patients who were clinically suspected for the nosocomial bloodstream, wound, and urinary tract infections were enrolled consecutively during the study period.

#### Data and specimen collection

Patients admitted in different wards including ICU were followed prospectively and assessed for the development of the surgical site, urinary tract, and bloodstream NI by internists and surgeons as per the European Centre for Disease Prevention and Control criteria [24]. Information on socio-demographic variables as well as potential risk factors of IN was collected from each patient by face-to-face interview using a structured questionnaire. For children, the respective guardian/caregiver was interviewed. Clinical data related to chronic diseases, hospitalization, admission ward type, and previous antimicrobial taking history were collected by reviewing the patient’s medical record and consulting the attending physician and surgeon. Clinical specimens such as urine, blood, and wound swabs were collected as soon as NI was suspected.

#### Blood sample collection and processing

Venous blood samples of 10 ml, 5ml, and 2ml were aseptically collected from adults, children, and neonates, respectively. The samples were collected in duplicate for each patient from a different site within a 30-minute difference [25]. The collected blood samples were inoculated directly to 5–10 ml Tryptic Soya broth medium (Oxoid, England) and transported immediately to Amhara public health institute’s microbiology laboratory, Dessie branch. The inoculated broth was incubated overnight aerobically at 35–37°C and inspected for bacterial growth daily. Gram stain was done from blood culture bottles that showed growth and sub-cultured onto blood agar (BA) and MacConkey (MAC) agar (Oxoid, England) plates. Inoculated plates were incubated aerobically at 35–37°C and examined for bacterial growth after 24 hr. However, those blood culture bottles which did not show growth were continuously monitored for the potential growth of pathogens until 7 days, and plates with no growth after 7 days of incubation were reported as negative [25].

#### Wound sample collection and processing

Wound/pus specimens were collected aseptically by sterile cotton swabs dipped in normal saline using Levine’s technique [26]. All collected wound specimens were labeled and transported to Amhara public health institute’s microbiology laboratory within 30 minutes by placing the swab into the sterile test tubes having 0.5 ml of sterile normal saline solution. The specimen was inoculated on blood and MacConkey agar plate and incubated aerobically at 35–37°C for 24–48 hrs. All positive cultures were identified by colony characteristics on the respective media [26, 27].

#### Urine sample collection and processing

Urinary tract infection suspected patients (non-catheterized) were instructed to collect 10 ml midstream clean-catch urine samples using a sterile wide-mouth container. The same amount of urine sample was transferred to a sterile container after cleansing the outlet of a catheter of catheterized patients. The collected urine was immediately transported to Amhara public health institute’s microbiology laboratory and inoculated on blood and MacConkey agar plate using the calibrated loop that measurers about 1μL. All inoculated agar plates were incubated aerobically at 35–37°C for 24–48 hr and inspected for bacterial growth. Colonies on blood agar were counted using a colony counter and checked for significant bacteriuria. Cultures from catheterized and non-catheterized patients that grew ≥10^2^ CFU/ml and 10^5^ CFU/ ml, respectively were taken as significant bacteriuria and processed further [28].

### Identification of bacterial isolates

For heterogeneous colonies, sub-culturing of individual distinct colonies was performed to ensure pure cultures. All suspected *P*. *aeruginosa* and Acinetobacter isolates were identified phenotypically by manual standard microbiological methods based on colonial morphology, pigmentation of the colony, and cell morphology. Further characterization of the isolates was performed using biochemical tests including catalase, oxidase, urease, indole, citrate utilization, lysine decarboxylation, motility, glucose, and lactose fermentation tests [25].

### Antimicrobial susceptibility test

A modified Kirby Bauer disk diffusion method was used to test each isolate for in vitro antimicrobial susceptibility pattern based on the 2020 Clinical and Laboratory Standards Institute (CLSI) criteria [29]. About 3–5 freshly grown pure colonies were taken and a homogeneous inoculums suspension was prepared using sterile normal saline and adjusted to 0.5 McFarland standard’s turbidity. The sterile cotton swab was dipped into the prepared suspension, rotated several times, and swabbed over the entire surface of the Mueller Hinton agar plates. Antimicrobial impregnated paper disks were placed on the inoculated plates. Both *Acinetobacter spp*. and *P*. *aeruginosa* isolates were tested against ceftazidime (30μg), ciprofloxacin (5μg), gentamicin (10μg), meropenem (10μg), piperacillin-tazobactam (100/10μg) and amikacin (30μg). Additionally, *Acinetobacter* isolates were tested against cefotaxime (30μg) and trimethoprim-sulfamethoxazole (1.25/23.75μg), while *P*. *aeruginosa* isolates were tested against aztreonam (30μg). *P*. *aeruginosa* isolate inoculated plates with respective antimicrobials were incubated aerobically at 35˚C for 16 to 18 hr, whereas the incubation period was 20-24hr for *Acinetobacter* isolates. The zone of inhibition was measured by calibrated ruler and interpreted as sensitive, intermediate, or resistant based on the CLSI guidelines [29]. However, for the description of the MDR profile, intermediate results were categorized as resistant. An isolate was considered MDR if it is resistant to at least one agent in three or more antimicrobial categories [30].

### Quality assurance

The questionnaire was pre-tested for its quality and validity before data collection. Data collectors were trained on the data collection procedure and interview techniques. The standard operating procedure was strictly adhered and quality control measures were implemented throughout the whole laboratory process. Culture media was prepared according to the manufacturers’ instructions. The sterility of prepared culture media was checked by incubating 5% of the batch at 35–37°C overnight before using it. Sample collection as well as bacterial isolation, identification, and antimicrobial susceptibility testing were performed in strict aseptic conditions. Media performance and potency of antimicrobial discs were tested using American Type Culture Collection (ATCC) standard reference strains (*P*. *aeruginosa* ATCC27853 and *E*. *coli* ATCC 25922). A 0.5% McFarland turbidity standard was used to standardize bacterial inoculums suspension for the antimicrobial susceptibility testing.

### Data analysis

Collected data were checked for their completeness and entered into Epi-Data version 3, then exported to Statistical Package for Social Sciences (SPSS) version 23 for further data cleaning and analysis. Descriptive statistics were computed and presented using words and tables. Bivariable and multivariable analyses were computed to identify factors independently influencing the occurrence of dependent variables. The odds ratio and 95% confidence interval were calculated to measure the strength of the association. A P-value of <0.05 was considered statistically significant.

#### Ethics approval and consent to participate

The protocol was ethically approved by Wollo University, College of Medicine, and Health science ethical review committee. To access the data, permission was obtained from Dessie Comprehensive Specialized Hospital. We followed all chains of command to `get support from legally authorized representatives for data collection. After clarifying the objective of the study, written informed consent was obtained from adult study participants; moreover, the ascent was obtained from guardians or caregivers of study participants whose age is below 18 years. Confidentiality of the result was maintained anonymously and used only for the study purpose. Positive findings were communicated to the attending clinician for appropriate treatment.

## Results

### Socio-demographic and clinical characteristics of study participants

A total of 254 patients with clinical evidence of nosocomial infection were enrolled in this study. The majority of the study participants were males, 145 (57.1%). The age of the study participants ranged from 0 to 90 years with a mean age of 29.8 years with a standard deviation of ± 24.3. The majority (35%) of participants were found in the age group of 31–60 years. One hundred fifty-seven (61.8%) study participants were living in rural settings. About 33% of study participants were illiterate in educational status. Similarly, 25.95% of study participants were children under age to have specific occupations followed by farmers, 60(23.62) (Table 1).

**Table 1:** Demographic and clinical characteristics of patients clinically suspected for nosocomial infection at Dessie comprehensive specialized hospital, Amhara region, North East Ethiopia, 2020.

Demographic and clinical variables		Frequency	Percentage (%)
Sex	Male	145	57.1
	Female	109	42.9
Age in years	0–15	88	34.6
	16–30	47	18.5
	31–60	89	35
	>60	30	11.8
Residence	Rural	157	61.8
	Urban	97	38.2
Education status	Primary	59	23.2
	Illiterate	91	35.8
	Secondary and above	40	15.7
	Under age (NA)	64	25.2
Occupation	House wife	54	21.26
	Employed	13	5.1
	Farmer	60	23.62
	Merchant	9	3.5
	Daily laborer	7	2.8
	Not applicable	66	25.98
	Student	35	13.77
	**Others	10	3.93
Patient admission location	Medical	86	33.9
	Surgical	27	10.6
	ICU	58	22.8
	Pediatric	21	8.3
	Orthopedics	52	20.5
	Gynecology	10	3.9
History of the previous admission	Yes	61	24.02
	No	193	75.98
Underlying chronic disease	Yes	80	31.49
	No	174	68.50
Previous antimicrobial taking history	Yes	184	72.44
	No	70	27.55
Type of antimicrobials taken at admission	One	26	10.2
	Two	76	29.9
	Three	26	10.2
	More than three	25	9.8
	Not known	31	12.2
	No antimicrobials taken	70	27.6
Associated invasive devise during admission	Intravenous cannula	178	70.07
	Urinary catheter	17	6.69
	Intravenous cannula and urinary catheter	38	14.96
	*Others	10	3.93
	No invasive device	11	4.33

**Key:** NA = Not applicable ‘

*Others = S-fix(n = 7), Pin(n = 3)

**Others = Pensioner (n = 2), no occupation(n = 8)

The majority of study participants were from medical wards (33.9%) followed by ICU (22.8%). About 3/4^th^ of study participants had no history of previous admission and 68.5% of participants do not have an underlying chronic disease. However, 72.44% of participants had taken different types of antimicrobials during admission and 178(70.07%) participants utilized intravascular catheters during their medication as shown in Table 1.

### Rate of *Acinetobacter spp*. and *P*. *aeruginosa* nosocomial infection

From a total of 254 collected clinical samples, *Acinetobacter* spp. and *P*.*aeruginosa* were isolated from wound/pus and blood. All of the urine specimens did not show significant bacterial growth (colony counts less than 10^2^ or 10^5^ CFU for catheterized and non-catheterized patients, respectively). A total of 34 bacterial isolates were isolated with an overall culture positivity rate of 13% (33/254). The proportion of bloodstream infection and wound infection was 13.33% (10/75) and 23.23% (23/99), respectively. From the total of 34 isolates, 70.6% were isolated from patients who developed wounds, with an equal number of *Acinetobacter* spp. and *P*. *aeruginosa*. The proportion of isolated *P*. *aeruginosa* (17.6%) was more among patients with bloodstream infection compared to *Acinetobacter* spp. (11.8%). All *Acinetobacter* spp. and about 78% of *P*. *aeruginosa* isolates were from patients with intravenous catheterization as shown in Table 2.

**Table 2:** Distribution of *Acinetobacter* spp and *P*. *aeruginosa* isolated from patients clinically suspected for nosocomial infection at Dessie comprehensive specialized hospital, Amhara region, North East Ethiopia, 2020.

		Culture result			Isolated bacteria		
		Negative	Positive	Total			
		n(%)	n(%)	n (%)			
					*Acintobacter* spp.; n(%)	*P*. *aeruginosa;* n(%)	Total
							n(%)
Type of clinical specimens							
	Pus/wound	76(29.9%)	23(9.1%)	99(38.9%)	12(35.3%)	12(35.3%)	24 (70.6%)
	Blood	65(25.6%)	10(3.9%)	75(29.5%)	4(11.8%)	6(17.6%)	10 (29.4%)
	Urine	80(31.5%)	0(0%)	80(31.5%)	-	-	-
Total		221(87%)	33(12.9%)	254(100%)	16(47.1%)	18(52.9%)	34(100%)
Type of associated invasive devise							
	Intravenous canula	149(58.7)	29(11.4%)	178(70.1)	16(47.1%)	14(41.2%)	30(88.2%)
	Urinary catheter	16(6.3%)	1(0.4%)	17(6.7)	0	1(2.9%)	1(2.9%)
	Intravenous canula and urinary catheter	38(14.96	0(0%)	38(14.96)	-	-	-
	Others*	7(2.8)	3(1.2%)	10(3.9)	0	3(8.8%)	3(8.82%)
	No associated device	11(4.3)	0(0%)	11(4.3)	-	-	-
Total		221(87)	33(12.99)	254(100)	16(47.1)	18(52.9)	34(100)

Key

*Others = S-fix, Pin

### Factors associated with nosocomial infection

Exploring independent risk factors of NIs showed that type of admission ward, number of antimicrobials taken at admission, and having chronic diseases/infections were independent predictors of NI due to either of *Acinetobacter spp*. and/or *P*. *aeruginosa*. Patients admitted in the surgical ward (AOR: 10.66; 95%CI: 1.22–93.23), Pediatric ward (AOR: 14.37; 95% CI: 1.4–148.5), ICU (AOR: 41.93; 95%CI: 4.7–374.7) and orthopedics (AOR: 52.21;95%CI: 7.5–365) were significantly at risk to develop NI as compared to patients admitted in the medical ward. Admitted patients having an underlying chronic disease such as HIV (AOR: 7.70; 95%CI:1.33–44.75), diabetes (AOR: 8.84; 95%CI:1.03–75.66), and others (Tuberculosis, Cardiac and Neurological disease) were statically significantly at risk of NI compared to admitted patients who had no underlying chronic disease. On the other hand, patients who took three and more antimicrobial types during admission were 94% times more protected from NI as compared to patients who did not take any antimicrobial as shown in Table 3.

**Table 3:** Association of independent variables with either of *Acinetobacter* spp. and/or *P*. *aeruginosa* infection among patients clinically suspected for nosocomial infection at Dessie comprehensive specialized hospital, Amhara region, North East Ethiopia, 2020.

		*Acinetobacter* spp. and/or *P*. *aeruginosa*		p. value	COR(CI)	p. value	AOR(CI)
Demographic and clinical variables		No	Yes				
Sex	Male	123(84.8)	22(15.2)	ref			
	Female	98(89.9)	11(10.1)	0.24	1.59(0.74–3.4)		
Age in years	0–15	74(84.1)	14(15.9)	0.10	5.5(0.69–43.6)		
	16–30	43(91.5)	4(8.5)	0.38	2.7(0.29–25.37)		
	31–60	75(84.3)	14(15.7)	0.11	5.4(0.68–43.05)		
	>60	29(96.7)	1(3.3)	ref			
Education status	Illiterate	80(87.9)	11(12.1)	ref			
	Primary school	53(89.8)	6(10.2)	0.72	0.82(0.29–2.36		
	Secondary school and above	33(82.5)	7(17.5)	0.41	1.54(0.55–4.32		
	Under age (NA)	55(85.9)	9(14.1)	0.72	1.19(0.46–3.06		
Residence	Rural	138(87.9)	19(12.1)	0.59	0.82(0.39–1.71)		
	Urban	83(85.6)	14(14.4)	ref			
Occupation	Employed	12(92.3)	1(7.7)	ref			
	Unemployed	209(86.7)	32(13.3)	0.565	1.84(0.23–14.6)		
Antimicrobial history	No	62(88.6)	8(11.4)	ref			
	Yes	159(86.4)	25(13.6)	0.648	1.2(0.52–2.85)		
Previous drug use length	1–7 days	100(90.1)	11(9.9)	ref			
	No described	60(89.6)	7(10.4)	0.908	0.94(0.5–2.56)		
	8–15 days	24(82.8)	5(17.2)	0.360	1.79(022–6.18)		
	>15 days	37(78.7)	10(21.3)	0.117	2.32(0.8–6.62)		
Admission ward	Medical	84(97.7)	2(2.3)	ref	-	Ref	
	Surgical	24(88.9)	3(11.1)	0.078	5.25(0.83–33.2)	0.032	10.66(1.22–93.23)
	ICU	49(84.5)	9(15.5)	0.011	7.71(1.6–37.16)	0.001	41.93(4.7–374.7)
	Paediatric	18(85.7)	3(14.3)	0.040	7.0(1.1–44.97)	0.025	14.37(1.4–148.5)
	Orthopaedics	37(71.2)	15(28.8)	0.001	17.027(3.7–78.3)	0.001	52.21(7.5–365)
	Gynaecology	9(90)	1(10)	0.227	4.67(0.38–56.68)	0.061	16.48(0.88–307.7)
History of Previous admission	No	168(87.0)	25(13.0)	ref			
	Yes	53(86.9)	8(13.1)	0.97	1.01(0.43–2.38)		
Duration of hospital admission	2–7 days	151(90.4)	16(9.6)	ref			
	>7 days	70(80.5)	17(19.5)	0.028	2.29(1.09–4.8)		
Number of antimicrobials taken at admission	1 drug type	24(92.3)	2(7.7)	0.597	0.646(0.13–3.26)	0.298	0.27(0.02–3.18)
	2 drug types	62(81.6)	14(18.4)	0.242	1.750(0.69–4.47)	0.421	0.44(0.06–3.28)
	3 drug types	24(92.3)	2(7.7)	0.597	0.646(0.13–3.26)	0.036	0.06(0.004–0.84)
	>3 drug types	24(96)	1(4)	0.299	0.323(0.04–2.722	0.048	0.06(0.004–0.97)
	Not known	25(80.6)	6(19.4)	0.293	1.860(0.59–5.90)	0.610	0.66(0.13–3.33)
	No drug taken	62(88.6)	8(10.4)	ref	-	Ref	
Underlying chronic disease	Kidney disease	14(87.5)	2(12.5)	0.993	0.99(0.21–4.67)	0.167	3.84(0.57–25.88)
	Diabetes	12(85.7)	2(14.3)	0.853	1.16(0.24–5.53)	0.047	8.84(1.03–75.66)
	Hypertension	27(96.4)	1(3.6)	0.194	0.26(0.03–1.99)	0.478	2.42(0.21–27.88)
	HIV	12(75)	4(25)	0.176	2.32(0.69–7.83)	0.023	7.70(1.33–44.75)
	Others	3(60)	2(40)	0.103	4.63(0.73–29.32)	0.030	15.49(1.3–184.59)
	None	153(87.4)	22(12.6)	ref	-	Ref	

Keys: Others = TB, Cardiac disease, Neurological disease

### Antimicrobial susceptibility profiles

The antimicrobial susceptibility profiles of the isolated *Acinetobacter* spp. and *P*. *aeruginosa* are shown in Table 4. Of the total isolates, 85.3% and 73.5% were resistant to ceftazidime and piperacillin-tazobactam respectively, whereas 11.8% of isolates were resistant to amikacin. *Acinetobacter* spp. showed more resistance rate to cefotaxime (93.8%), piperacillin-tazobactam, and ceftazidime (87.5% for each), but the least resistance rate was observed for amikacin (12.5%) followed by ciprofloxacin (18.8%). Similarly, *P*. *aeruginosa* isolates were more resistant to ceftazidime (83.3%) and aztreonam (77.8%); however, 88.9% and 83.3% of *P*. *aeruginosa* isolates were susceptible to amikacin and meropenem, respectively.

**Table 4:** Antimicrobial resistance pattern of *Acinetobacter* spp. and *P*. *aeruginosa* isolated from patients clinically suspected for nosocomial infection at Dessie comprehensive specialized hospital, Amhara region, North East Ethiopia, 2020.

Isolates	tested	CTX	AN	TZP	CIP	CAZ	GM	MEM	COT	ATM
		r(%)	r(%)	r(%)	r(%)	r(%)	r(%)	r(%)	r(%)	r(%wwr)
*Acinetobacter* spp.	16	15(93.8)	2(12.5)	14(87.5)	3(18.8)	14(87.5)	8(50)	7(43.8)	11(68.8)	NA
*P aeruginosa*	18	NA	2(11.1)	11(61.1)	11(61.1)	15(83.3)	5(27.8)	3(16.7)	NA	14(77.8)
Total	34	15(44.1)	4(11.8)	25(73.5)	14(41.2)	29(85.3)	13(38.2)	10(29.4)	10(68.8)	14(77.8)

**Key:** r(%)—number and Percentage of resistant isolates; NA- Not applicable; CTX-Cefotaxime 30μg, AN-Amikacin 30μg, TZP-Piperacillin-tazobactam 100/10 μg, CIP- ciprofloxacin 5μg, CAZ- ceftazidime 30μg, GM- Gentamicin 10μg, MEM- Meropenem 10μg, COT-Trimethoprim sulfamethoxazole 1.25/23.5μg, ATM- Aztereonam 30 μg.

#### Multidrug-resistance profiles of isolates

The overall MDR rate of isolated bacteria was 82.35%. About 81% of *Acinetobacter* spp. and 83% of *P*. *aeruginosa* isolates showed MDR. Among MDR isolates one and four isolates of each *Acinetobacter* spp and *P*. *aeruginosa* were resistant against antimicrobials from 6 and 5 different categories, respectively (Table 5).

**Table 5:** Multi-drug resistance profile of *Acinetobacter* spp. and *Pseudomonas* aeruginosa isolated from patients clinically suspected for nosocomial infection at Dessie comprehensive specialized hospital, Amhara region, North East Ethiopia, 2020.

	Antibiogram profile	Number of resisted antimicrobial categories	Resistance level	Number of Isolates (%)
***Acinetobacter* spp. (n = 16)**	CTX, AN, TZP, CAZ, GM, MEM, COT	5	MDR	1/6.25
	CTX, TZP, CIP, CAZ, GM, MEM, COT	6	MDR	1/6.25
	CTX, AN, TZP, CAZ, GM, MEM	4	MDR	1/6.25
	CTX, TZP, CIP, CAZ, GM, COT	5	MDR	2/12.5
	CTX, TZP, CAZ, GM, MEM, COT	5	MDR	1/6.25
	CTX, TZP, CAZ, GM, COT	4	MDR	1/6.25
	CTX, TZP, CAZ, MEM, COT	4	MDR	2/12.5
	CTX, TZP, CAZ, GM	3	MDR	1/6.25
	CTX, TZP,CAZ,MEM	3	MDR	1/6.25
	CTX, TZP, CAZ, COT	3	MDR	1/6.25
	CTX, TZP, COT	3	MDR	1/6.25
	CTX, TZP, CAZ	2	Not MDR	1/6.25
	CTX, CAZ, COT	2	Not MDR	1/6.25
**Total percentage of MDR isolates**				**13(81.25)**
*P*. *aeruginosa* (n = 18)	TZP, CAZ, CIP, GM, MEM, ATM	6	MDR	1/5.55
	AN, TZP, CAZ, CIP, GM, ATM	5	MDR	1/5.55
	TZP, CAZ, CIP, GM, ATM	5	MDR	2/11.11
	TZP, CAZ, CIP,MEM, ATM	5	MDR	1/5.55
	TZP, CAZ, CIP, ATM	4	MDR	2/11.11
	TZP, CAZ, GM, ATM	4	MDR	1(5.55)
	TZP, CIP, ATM	3	MDR	1(5.55)
	AN,CIP,CAZ	3	MDR	1(5.55)
	TZP, CAZ,ATM	3	MDR	2(11.11)
	CIP,CAZ, ATM	3	MDR	2(11.11)
	CAZ,MEM,ATM	3	MDR	1(5.55)
	**Total percentage of MDR isolates**			**15(83.3)**
**Total percentage of MDR isolates**				**15 (83.33)**

**Key:** CTX-Cefotaxime, AN-Amikacin, TZP-Piperacillin tazobactam, CIP- ciprofloxacin, CAZ- ceftazidime, GM- Gentamicin, MEM- Meropenem, COT-Trimethoprim sulfamethoxazole, ATM- Aztreonam

## Discussion

Even though NI is a global health threat, it is alarmingly increasing in low-income countries like Ethiopia with a national prevalence rate of 16.96% [4]. Different bacterial species are associated with NI [11, 13, 31, 32], and the high rate of drug-resistant nosocomial bacteria poses a significant threat to the infected patients, communities, and health care providers [13]. *A*. *baumannii* and *P*. *aeruginosa* are known to develop resistance to most antibiotics. NIs due to MDR *A*. *baumannii* and *P*. *aeruginosa* is a critical health problem to hospitalized patients [23].

In the present study, the prevalence of combined *Acinetobacter* spp. and *P*. *aeruginosa* infection was 13% (33/254) which is consistent with a previous report from India (14.8%) [33]. However, it was relatively higher than reports from Ethiopia (1.6–8.4%) [13, 14, 23, 32], Iran (0.4–0.7%) [12, 34], Italy (9.3%) [35] and Uganda (8.2%) [36]. The rate of culture-confirmed *Acinetobacter* infection was 6.3% which is in agreement with a study conducted in India (5.97%) [33]. In contrast, the current result is higher than previous reports from Ethiopia (0.5–3.8%) [14, 23], Iran (0.2–0.4%) [12, 34] and Uganda (3%) [36], and lower than reports from Morocco (8.4%) [37] and Uganda (9.9%) [38]. Similarly, we found a 7.1% prevalence of *P*. *aeruginosa* infection and this is similar to previous reports in India (6.8%) [39]. In contrast, our result is relatively higher than reported by Sharma et al (8.9%) [33] in India and Ahmed et al (19%) [40] in Egypt, while lower than reported in Ethiopia (1% to 4.7%) [13, 14, 23, 31, 32] and elsewhere in the world (0.2% to 5.4% [12, 34, 36–38]. The possible explanation for the observed difference might be due to variation in sample size, clinical site of NI, the severity of underlying diseases, hospital settings, patients’ exposure to different invasive medical devices, standards of infection prevention practice, and length of hospitalization. Moreover, the observed high prevalence of combined *Acinetobacter* and *P*. *aeruginosa* associated NI might be due to the commonly observed overcrowding of patients, poor infrastructure of the hospital, and poor implementation of infection control measures especially hand hygiene practices and decontamination of the hospital environment.

The rate of NIs was significantly higher among patients with underlying chronic diseases like HIV and diabetes as shown in Table 3. This can be explained because patients with chronic underlying diseases, besides immune suppression, could visit health facilities frequently and they are potentially at risk to be contaminated with bacteria circulating in the health facilities as a result develop nosocomial infection even with low virulent pathogens. This is evidenced from a previous study that revealed *Acinetobacter* spp NIs was significantly associated with immunosuppression [41]. Our result also showed that patients admitted in the surgical ward, pediatric, ICU, and orthopedics were significantly at risk to develop NIs (Table 3) which is consistent with a previous study conducted in France [42]. Acinetobacter-associated NIs is found significantly associated with admission to the ICU [41]. A recent systematic review and meta-analysis revealed that the rate of NIs was more in the ICU followed by the pediatric ward, surgical ward, and obstetrics ward [4]. This might be linked with poor infection prevention practices observed in some health facilities [43] and the high rate of exposure of patients to nosocomial pathogens from the hospital environment including health care professionals. But, our study hospital’s infection prevention practice level is not well known and needs immediate investigation.

Patients who took more than three antimicrobial types at the time of admission were 94% times more protective from nosocomial infection as compared to patients not taking antimicrobials at all. In contrast, Uwingabiye et.al indicated that previous exposure to antibiotic polytherapy was a risk for ICU acquired *A*. *baumannii* infection but the classes of taken antibiotics are not specified [37]. Playford et.al also showed that previous exposure of patients to antibiotics is a significant risk factor for *A*. *baumannii* infection [44]. On the other hand, taking active antipseudomonal antibiotics is described as protective [45], but taking inactive antibiotics has been identified as a risk for NIs [46]. In contrast, Hoang et.al found no significant association with inactive antibiotic support and *P*. *aeruginosa* infection, but there is an association with colonization [42]. In our result, the observed protective role of previous antibiotic polytherapy might be due to the incorporation of active antibiotics and their synergistic effect. However, we are not able to describe the types of antibiotics taken by patients and this could be a limitation of this study. Thus, the role of number and type of antibiotic therapy for the prevention of NI warranted further investigation.

In the present study, resistance was observed to different antibiotics. *Acinetobacter* isolates were highly resistant to cefotaxime (93.8%) and piperacillin-tazobactam (87.5%). Similarly, a 90% resistance rate was observed against piperacillin-tazobactam in Vietnam [47], China [48], and Turkey [49]. In contrast, a low resistance rate was reported against piperacillin-tazobactam in Uganda (55%) [36] and Iran (24%) [34]. Moreover, 87.5% of isolated *Acinetobacter* species was resistant to ceftazidime which is in agreement with previous reports from Ethiopia (77.8%) [23], Uganda (80%) [38], Vietnam (90%) [47], China (92.6) and Turkey (86.7%) [49]. But, our result is higher than reports from Iran (15%) [34] and Uganda (45%) [36]. Another recent study conducted in Iran also reported a higher prevalence (96.6%) of ceftazidime resistant *A*. *baumannii* [50]. Similarly, 83.3% and 77.8% of *P*. *aeruginosa* isolates were resistant to ceftazidime and aztreonam respectively which is comparable to previous reports with a ceftazidime resistance rate of 100% in Ethiopia [23], 69% in Uganda [36], 77.7% in India [39] and 72.4% in Vietnam [47]. In contrast, less number of aztreonam (40%) and ceftazidime (50%) resistant *P*. *aeruginosa* isolates were reported in Uganda [36] and Iran [34] respectively. The observed differences might be due to variations in resistance screening methods and antimicrobial prescription policy and frequency. Furthermore, the variation could be due to genetic alteration of isolates caused by unnecessarily prescribed antibiotics in different countries around the world [51]. In the current study, the highest level of resistance against cefotaxime, ceftazidime, piperacillin-tazobactam, and aztreonam might be linked with excessive and inappropriate use of these antibiotics in the study area since there is no clear antibiotic policy and controlling mechanisms of antimicrobial usage in Ethiopia.

In the present study, *Acinetobacter* spp. showed 18.8% resistance rate to ciprofloxacin which is lower than previous findings reported in Ethiopia (44.5%) [23], Iran (68–97.4%) [34, 50], Uganda (62–88%) [36, 38], Vietnam (91%) [47], China (92.6%) [48] and Turkey (53.3%) [49]. We also found that the resistance rate of *P*. *aeruginosa* to ciprofloxacin and gentamicin was 61.1% and 27.8%, respectively. A relatively similar ciprofloxacin-resistant rate was found in Iran (52%) [34] and Uganda (50–64%) [36, 38]. The currently observed gentamicin resistance rate was in agreement with a study conducted in Iran (28%) [34]. But more gentamicin resistant *P*. *aeruginosa* isolates were reported in Uganda (69%) [36] and Ethiopia (54.55) [23]. In contrast to our result, Motbainor et.al showed that 36.4% of *P*. *aeruginosa* isolates were resistant to ciprofloxacin in Ethiopia [23].

In this study, MDR was observed in 28 isolates (82.4%), which is extensively high. The proportion of MDR isolates of *Acinetobacter* spp. and *P*. *aeruginosa* was 81.3% and 83.3% respectively. Previously in Ethiopia, scholars reported that all nosocomial isolates of *A*. *baumannii* and *P*. *aeruginosa* were MDR [14, 23]. The currently observed proportion of MDR *P*. *aeruginosa* isolates was in agreement with previous reports from Uganda (81%) [36] and India (84.7%) [39], but higher than Egypt (52%) [40] and Northeast of Iran (16.5%) [50]. And also the proportion of MDR isolates of *Acinetobacter* spp. was higher than reports from Uganda (62%) [36] but comparable to reports from northeast Iran (74.75%) [50]. Differences in the selected panel of antimicrobial agents could be the possible explanations for variations in the prevalence of MDR between the present study and other similar studies carried out elsewhere. Moreover, the observed high rate of MDR isolates could be associated with their biological natures. They have been known to express different antimicrobial resisting mechanisms including the production of aminoglycoside modifying enzymes, ESBLs, carbapenemase, topoisomerases as well as outer membrane proteins as penicillin-binding proteins [2, 17, 18].

Our study revealed a low level of amikacin-resistant *Acinetobacter* (12.5%) and *P*. *aeruginosa* (11.1) isolates. Previously in Iran [34] and Uganda [36], a low proportion of amikacin-resistant *Acinetobacter* isolates (<25%) were reported. In contrast, Özdemir et.al in Turkey reported that *Acinetobacter* isolates resistant to amikacin was 86% [49]. In Egypt, Mahmoud et.al found amikacin is effective against nosocomial *P*. *aeruginosa* isolates [40], but more proportion of amikacin-resistant isolates were reported in Ugandan (60%) [38] and Vietnam (65.5%) [47]. In the current study, meropenem is most effective against *P*. *aeruginosa* isolates with a resistance rate of 16.7%, which is supported by a study conducted by Sharma et.al [33]. However, 45.5% and 86% of previous isolates were resistant to meropenem in Ethiopia [23] and Vietnam [47] respectively. About 40% of *Acinetobacter* isolates were resistant to meropenem. Our result is in line with previous reports in Uganda (28–50%) [36, 38] and Turkey (53.3%) [49], but lower than previous reports from Vietnam (90%) [47] and China (93.6%) [48]. The currently observed relative effectiveness of meropenem and amikacin against *Acinetobacter* and *P*. *aeruginosa* might be due to the low level of prescription practice of these antimicrobials since they are used as the last treatment option for serious infections and comparatively more expensive in Ethiopia. Infections caused by MDR *Acinetobacter* and *P*. *aeruginosa* isolates could be treated with either meropenem or amikacin and more strongly using a combination of these antimicrobials. Recent literature also showed promising activity of carbapenem subclass of β-lactams against drug-resistant *Acinetobacter* spp and *P*. *aeruginosa* [52].

There are some limitations of the current study. This study assesses the point prevalence of NI in which a single sample is collected at a point in time. Further, patients with a problem of the respiratory system were not included due to patient quarantine and fear of coronavirus transmission. Secondly, being a cross-sectional study, hospital-acquired infections that arose after discharge were not detected due to lack of follow-up. These could underestimate the prevalence of NI as well as the isolation rate of investigated organisms. Third, we could not conduct a molecular analysis to identify bacterial species especially *Acinetobacter* species and this is a significant weakness of the study. Finally, the sample size for the study was humble and did not have adequate power to detect smaller effect sizes. Thus, these results should be interpreted and utilized cautiously.

## Conclusions

Our results showed an overall prevalence of 13.3% nosocomial infection with either *Acinetobacte*r spp. or *P*. *aeruginosa*. Underlying chronic diseases such as HIV and diabetes were significant associated factors for nosocomial infection. Admission in the surgical, Paediatric, ICU, and Orthopaedics wards were a significant factor for nosocomial *Acinetobacte*r species and *P*. *aeruginosa* infection. Even though, amikacin and meropenem showed promising activity against *Acinetobacte*r spp. and *P*. *aeruginosa* isolates, the proportion of MDR isolates of *Acinetobacter* spp and *P*. *aeruginosa* was 81.3% and 83.3% respectively. It is also concluded that treatment of NIs should be guided by antimicrobial susceptibility testing.

## Supporting information

S1 File (DOCX)

## Abbreviations

CFU: Colony Forming Unit
CLSI: Clinical and Laboratory Standards Institute
DCSH: Dessie comprehensive specialized Hospital
HCAI: healthcare-associated infection
HIV: human immunodeficiency virus
ICU: Intensive Care Unit
MDR: Multi-Drug Resistance
NI: Nosocomial Infection
NIs: Nosocomial Infections
OPD: outpatient department
SPSS: Statistical package for social science
WHO: world health organization
