# Supplementary material for: Antimicrobial resistance profiles and associated factors of Acinetobacter and Pseudomonas aeruginosa nosocomial infection among patients admitted at Dessie comprehensive specialized Hospital, North-East Ethiopia. A cross-sectional study
Source: PLoS One. 2021 Nov 15;16(11):e0257272. doi: 10.1371/journal.pone.0257272 (PMC8592406; doi:10.1371/journal.pone.0257272)
Supplement: S1 File — (DOCX) [file pone.0257272.s001.docx]

# Annex III: English Version Questionnaire

**Questionnaire**

Investigation of Antimicrobial resistance in *Acinetobacter* species, *Pseudomonas aeruginosa* and its associated factors among admitted patients at Dessie comprehensive specialized Hospital, North-Eastern Ethiopia.

| Questionnaire code #------------------------------- | Card #---------------------------------- |
| --- | --- |
| Date of data collection------------------------------ | Name of health institution-------------------- |

**I. Socio-demographic information**

| **SN** | **QUESTIONS** | **RESPONSE CATEGORIES** |
| --- | --- | --- |
| Q101 | Age( in years) ____________________ | |
| Q102 | Sex | Male---------------1 Female-------------2 |
| Q103 | Education status | No education-----1 Primary-------------2  Secondary--------3 Higher -----------4 |
| Q104 | Current residence | Rural-------------1 Urban--------------2 |
| Q105 | Occupation | Employed------------1 Farmer------------2  Merchant-------------3 House wife--------4  Daily labourer -----5 Other specify-------6 |
| **II .Risk factors for Acinetobacter and *Pseudomonas aeruginosa* infection**   \| **Code** \| **Question** \| **RESPONSE CATEGORIES** \| \| --- \| --- \| --- \| \| Q201 \| Have you been admitted in the hospital for treatment \| Yes------------1 No-----------2 \| \| Q202 \| If yes for Q#201, how long \| ---------------------- \| \| Q203 \| Is there any associated invasive devise during admission \| 1. No invasive devise 2. Intravenous canula 3. Urinary catheter 4. Intravenous canula and urinary catheter 5. Others --------------- \| \| Q204 \| Have you taken an antimicrobial treatment during admission? \| Yes-------1 No----------2 \| \| Q205 \| If yes for Q#204, number of antimicrobials taken at admission \| One------1 Two-------2  Three-----3 >Three-----4  Not known---5 \| \| Q206 \| If yes for Q#204, For how long? \| --------------------------------- \| \| Q207 \| Location of Patient/Ward \| Surgical-------1 Medical-----------2  ICU Adult-----3 ICU neonatal----4  Paediatric------5 Orthopaedics----6  Others specify------------7 \| \| Q208 \| Current reason for Hospital admission \| UTI------------------------------------1  Wound infection---------------------2  Blood stream infection--------------3 \| \| Q209 \| Do you have any of the following chronic disease \| Diabetes -------------------------------1  Neurological disease-----------------2  kidney disease------------------------3  Cardiac disease-----------------------4  Hypertension-------------------------5  HIV-------------------------------------6  Other specify-------------------------7  No.....................................................8 \| \| Q210 \| For how long have you been admitted for the current case? \| ------------------------------------day/s \| \| Q211 \| Type of clinical sample(s) \| -------------------------------------------- \| \| Q212 \| Result \| ---------------------------------------------- \| | | |

#

# Annex IV: Amharic Version of Questionnaire

**የአማረኛ መጠይቅ**

አሲኒቶባክትር እና ሲዶሞናኤሮጅነሳ የተባለውን ባክቴሪያ መለየትና ፀረ-ባክቴሪያ የተቋቋሙ መድሀኒቶችን በደሴ ሪፈራል ሆስፒታል ተኝተው በሚታከሙ ሕመምተኞች ቁስል፤ደም፤ሽንት ውስጥ መኖራቸውን መለየት እና በምን ያህል መጠን እንዳሉ ለማሳወቅ የተዘጋጀ መጠይቅ፡፡

| የኮድ ቁጥር------------------------- | የመታከሚያ ካርድ ቁጥር---------------- |  |
| --- | --- | --- |
| ናሙናዉ የተወሰደበተ ቀን------------------------- | የጤና ተቋሙ ስም------------------------- |  |

**ሀ. ማህበራዊናኢኮኖማዊ መረጃ**

| **ተ.ቁ** | **ጥያቄ** | **ኮድ** |
| --- | --- | --- |
|  | እድሜ | **--------------------** |
|  | ጾታ | ወ-------------1 ሴ----------------2 |
|  | የትምህርት ደረጃ | ያልተማረ-------1 የመጀመሪያ ሳይክል-----------2  የሁለተኛ ሳይክል--3 የከፍተኛ ትምህርት----------4 |
|  | መኖሪያ ቦታ | ገጠር-------1 ከተማ------------2 |
|  | ስራ | ተቀጣሪ ሰራተኛ-----1 ገበሬ------------2  ነጋዴ-------------3 የቤትእመቤት-----4  የቀን ስራ--------5 ሌላ ከሆነ ይግለጹ--------6 |

**ለ .ለበሽታው መባባስ አጋላጭ ሁኔታዎች**

| **ጥያቄ ቁጥር** | ጥያቄ | መለያ |
| --- | --- | --- |
| 1 | ላለፋት 6 ወራት ለህክምና ሆስፒታል ተኝተው ያውቃሉ? | አዎ----------1 አይደለም--------2 |
| 2 | ከተኙ ለምን ያህል ጊዜ? | -------------------- |
| 3 | የትኛውን Invasive device ተጠቅመዋል | 1. Intravenous canula  2.Urinary catheter 2. Intravenous canula and urinary catheter 3. Others 5. No invasive devise |
| 4 | ፀረ-ባክቴሪያ መደሃኒት ወስደው ያውቃሉ? | አዎ----------1 አይደለም--------2 |
| 5 | ወስደው ካወቁ የወሰዱአቸው መድሀኒቶች ብዛት ስንት ናቸው? | 1. አንድ 2. ሁለት 2. ሶስት 4. ከሶስት በላይ 3. አላስታውስም |
| 6 | ለምን ያህልጊዜ መደሃኒት ወሰዱ? | ---------------------------------- |
| 7 | ታካሚው የሚገኝበት ቦታ | ቀዶ ጥገና---------1 የውስጥ ደዌ--------2  አዋቂወች ጽኑ ህሙማን-------------3  የጨቅላ ህጻናት ጽኑ ህሙማን-----------4  የህጻናት-----5 የአጥንትና መገጣጠሚያ---6  ሌላ ይገለጽ-----------------------------------7 |
| 8 | ታካሚው የተኛበት ምክንያት | የሽንት ቧንቧ መታወክ -------------------1  የቁስል መመርቀዝ -------------------------2  የደም መበከል ህመም----------------------3 |
| 9 | በበሽታው ምክንያት ለስንት ቀን ተኙ | በቀን ይግለጹ------------------------ |
| 10 | ከሚከተሉት ስር የሠደዱ ወይም ለረጅም ጊዜ የቆዩ ህመም ውስጥ የትኛው አለበዎት | ስኳር ህመም-----1 የነርብ ህመም -----2  የጉበትህመም----3 የደም ግፊት ህመም----4  የኩላሊት ህመም-----5 HIV--------------6  ሌላ ካለ ይጥቀሡ------7 |
| 11 | የተወሰደው የናሙና አይነት | -------------------------- |
| 12 | ውጤት | --------------------------- |
